# Transcriptomic analysis reveals immune dysregulation and identifies key genes in ICU patients with severe ARDS

**DOI:** 10.3389/fimmu.2026.1777938

**Published:** 2026-05-11

**Authors:** Haili Hu, Denghui Dou, Ruiling Shang, Rongzhi Luo

**Affiliations:** 1 Department of Critical Care Medicine, Renmin Hospital, Hubei University of Medicine, Shiyan, China; 2 Department of Burns and Plastic Surgery, Renmin Hospital, Hubei University of Medicine, Shiyan, China

**Keywords:** ARDS, hub genes, immune imbalance, single-cell analysis, transcriptomics

## Abstract

**Objective:**

Acute respiratory distress syndrome (ARDS) is characterized by severe immune dysregulation, yet its molecular determinants remain poorly defined. This study aimed to delineate the immune imbalance landscape of ICU patients with ARDS and to validate the expression and potential functional relevance of candidate hub genes through *in vitro* experiments.

**Methods:**

Bulk transcriptomic datasets were merged and analyzed using differential expression, WGCNA, and machine learning approaches. Functional enrichment and cell deconvolution were assessed, followed by single-cell transcriptomic validation. *In vitro* experiments with LPS-induced THP-1 cells were performed to confirm candidate gene expression.

**Results:**

Combined analyses highlighted immune-related pathways and revealed marked alterations in innate and adaptive immune subsets. Two histone-related genes, H2BC4 and H2BC12, emerged as candidate hub genes with preferential expression in myeloid populations and inducible upregulation under inflammatory stimulation.

**Conclusion:**

This study provides novel insights into ARDS immunopathogenesis and identifies potential molecular targets that may inform future diagnostic and therapeutic strategies.

## Introduction

Acute respiratory distress syndrome (ARDS) is a severe and life-threatening condition that frequently affects patients admitted to intensive care units (ICUs) (1, 2). Despite advances in supportive care, the mortality rate of ARDS remains as high as 30–40%, making it one of the most challenging syndromes in critical care medicine (3). The pathogenesis of ARDS is complex, involving alveolar epithelial injury, disruption of the pulmonary endothelial barrier, and uncontrolled inflammatory responses that culminate in respiratory failure (4). However, the precise molecular mechanisms underlying ARDS remain incompletely understood, and effective pharmacological therapies are still lacking.

Accumulating evidence suggests that immune dysregulation plays a central role in the onset and progression of ARDS (5). Excessive activation of innate immune cells, such as neutrophils and monocytes, leads to the release of proinflammatory cytokines and cytotoxic mediators, amplifying lung injury (6). Conversely, impaired adaptive immune responses may further exacerbate the imbalance between immune activation and resolution, contributing to poor outcomes (7, 8). Although several studies have attempted to characterize immune-related signatures in ARDS, a comprehensive understanding of the molecular determinants driving this immune imbalance is still limited.

With the rapid advancement of high-throughput sequencing technologies, transcriptome profiling has emerged as a powerful approach to systematically dissect disease-associated molecular networks (9). Bioinformatics methods, including differential expression analysis, weighted gene co-expression network analysis (WGCNA), and machine learning algorithms, allow the identification of robust gene signatures with potential diagnostic and therapeutic relevance. Moreover, single-cell transcriptomics provides an unprecedented resolution to unravel the cellular heterogeneity of ARDS and to link the expression of key genes with specific immune cell subsets.

Therefore, this study sought to delineate the immune imbalance landscape of ICU patients with ARDS by exploring transcriptomic analyses with network and machine learning approaches. We further aimed to identify hub genes with strong immunological relevance and validate their expression patterns through single-cell sequencing and experimental assays. These findings are expected to provide novel insights into the immunopathogenesis of ARDS and inform potential therapeutic strategies.

## Materials and methods

### Data acquisition and preprocessing

Transcriptomic datasets of ARDS were obtained from the Gene Expression Omnibus (GEO) database, including GSE200847 (41 ARDS patients and 5 controls; tracheal aspirates), GSE163426 (32 ARDS patients and 5 controls; tracheal aspirates), and the single-cell dataset GSE200848. GSE200847 included 41 ARDS patients (11 hyperinflammatory, 30 hypoinflammatory) and 5 mechanically ventilated controls without pulmonary disease. The hyperinflammatory phenotype showed significantly higher SOFA scores, elevated IL-8, IL-6, and soluble TNF R1, and lower protein C levels compared to the hypoinflammatory phenotype. GSE163426 comprised 15 COVID-19 ARDS patients, 32 ARDS patients from other etiologies, and 5 mechanically ventilated controls without ARDS. COVID-19 ARDS patients had longer hospitalization and a higher proportion of Hispanic ethnicity compared to other-ARDS patients. All ARDS patients met the Berlin criteria; controls were intubated for neurologic indications without pulmonary disease. These datasets were selected based on the availability of raw count data, clear case–control design, and consistency in sequencing platforms (Illumina NovaSeq 6000), which ensured comparability and reliability for integrative analysis. Raw expression matrices were first imported using the read.delim function in R. For duplicated gene symbols, expression values were averaged using the dplyr package. The first column of each dataset was unified as “gene”, and common genes between datasets were identified using the intersect function. The datasets were then merged based on shared genes using the merge function (by = “gene”, all = FALSE). Subsequently, log2(x+1) transformation was applied to stabilize variance and approximate normal distribution. All bulk RNA-sequencing datasets were generated on the Illumina NovaSeq 6000 platform. Raw count matrices were merged and log2(x+1) transformed. Batch effects were assessed by principal component analysis (PCA) and corrected using the ComBat function in the sva package, with post-correction PCA confirming effective removal. Quality control was performed to assess low-abundance genes and potential outliers; however, no samples were excluded based on these criteria.

### Differentially expressed gene analysis

Differential expression analysis between ARDS and control groups was performed using the DESeq2 package. Genes with |log2 fold change| > 1 and p < 0.05 were considered significantly differentially expressed. Volcano plots were generated to visualize the distribution of upregulated and downregulated genes in each dataset. To increase robustness, intersecting DEGs across the two datasets were identified as a conservative strategy to ensure reproducibility across cohorts and retained for subsequent analyses.

### WGCNA

Weighted gene co-expression network analysis (WGCNA) was performed using the WGCNA package in R. Gene expression data from GSE200847 and GSE163426 were merged after batch correction using the ComBat function in the “sva” package. Genes with low variability were filtered using a standard deviation threshold (SD > 0.5). The goodSamplesGenes function was used to detect missing values, and hierarchical clustering was performed to assess sample integrity. No samples were identified as outliers for exclusion. Module membership (MM) and gene significance (GS) were calculated, and their relationships were visualized to identify biologically relevant genes within key modules. A soft-thresholding power was selected based on the scale-free topology criterion (R² ≈ 0.90), resulting in an optimal β value of 10. A signed network was constructed, and modules were identified using hierarchical clustering and dynamic tree cutting (deepSplit = 2, minClusterSize = 30). Modules with high similarity were merged at a threshold of 0.25. Module-trait relationships were evaluated using Pearson correlation between module eigengenes and clinical traits, and p-values were adjusted using the Benjamini-Hochberg method. In this study, ARDS clinical status was defined as a binary variable (ARDS = 1, control = 0). Although the original dataset included ARDS subphenotypes (e.g., hyperinflammatory and hypoinflammatory), these phenotype labels were not included as traits in the WGCNA analysis.

### Machine learning–based identification of hub genes

To refine candidate hub genes, two machine learning algorithms were employed. In this study, hub genes were defined as genes consistently selected by both machine learning algorithms as key features associated with the ARDS clinical status. First, least absolute shrinkage and selection operator (LASSO) regression was applied to the intersecting gene set using the glmnet package, and the optimal lambda parameter was determined via leave-one-out cross-validation (LOOCV). Second, support vector machine–recursive feature elimination (SVM-RFE) was performed with the e1071 package, and genes with high importance scores were retained. The intersection of genes identified by both methods was considered robust hub gene candidates. It should be noted that, given the relatively small sample size, the machine learning models were not intended for predictive classification but rather for feature selection. Therefore, genes identified through LASSO and SVM-RFE were considered as candidates with potential biological relevance, rather than as optimal predictors for clinical diagnosis. The intersection of both methods was used to enhance robustness and reduce false positives.

### Functional enrichment analysis

Gene Ontology (GO) and Kyoto Encyclopedia of Genes and Genomes (KEGG) pathway enrichment analyses were performed on the 245 genes from the WGCNA darkseagreen4 module using the clusterProfiler package. Enrichment significance was assessed using a hypergeometric distribution test (also known as Fisher’s exact test), and significantly enriched terms were defined as those with p < 0.05 after Benjamini–Hochberg correction. Bubble plots were generated to visualize functional categories, with particular emphasis on immune-related biological processes and signaling pathways relevant to ARDS pathogenesis.

### Immune infiltration analysis

The merged expression matrix was input into the CIBERSORT algorithm to estimate the proportions of 22 immune cell types using the LM22 signature matrix. Relative immune cell fractions were compared between ARDS and control groups using Wilcoxon rank-sum tests. To account for multiple comparisons across the 22 immune cell types, p-values were adjusted using the Benjamini-Hochberg (BH) method, and adjusted p-values (FDR) < 0.05 were considered statistically significant. Results were visualized through stacked bar plots to display the overall immune landscape and boxplots to highlight significantly altered immune subsets.

### Single-cell transcriptome validation

The single-cell RNA-seq dataset GSE200848, comprising eight ARDS patient samples, was processed using the Seurat package in R. After quality control, low-quality cells with fewer than 200 detected genes or with high mitochondrial gene content exceeding 10% of total UMIs were removed. The remaining high-quality cells were normalized, and highly variable genes were identified. Dimensionality reduction was performed using principal component analysis (PCA), followed by Uniform Manifold Approximation and Projection (UMAP) for visualization of cellular heterogeneity. Cell clusters were annotated using a dual approach: first, automated annotation was performed with the SingleR package (reference-based); second, annotation was manually validated based on canonical lineage markers. In cases where discrepancies between SingleR and marker-based annotation were observed (approximately 5% of clusters, typically involving transitional or low-abundance populations), cluster identity was determined by consensus after manual review of top differentially expressed genes and canonical marker expression. Expression levels of candidate hub genes were extracted from the annotated clusters, and violin plots and feature plots were generated to display their distribution across immune cell subsets.

For visualization of gene expression on UMAP, Seurat::Feature plots were generated using default Seurat parameters without enabling the order = TRUE option, which may affect visualization of highly expressed cells.

### Modeling and grouping

The human peripheral blood monocyte cell line THP-1 (ATCC, catalog no. TIB-202) was used to establish an *in vitro* model of ARDS-related inflammation. Cells were cultured in RPMI-1640 medium supplemented with 10% fetal bovine serum (FBS) and 1% penicillin–streptomycin under standard conditions (37 °C, 5% CO^2^). To induce an inflammatory phenotype, THP-1 cells were stimulated with lipopolysaccharide (LPS, 1 µg/mL) for 6 h, a procedure widely used to mimic the excessive immune activation observed in ARDS (10). Cells maintained under identical culture conditions without LPS stimulation were used as the reference.

### Enzyme-linked immunosorbent assay

After LPS treatment, culture supernatants were harvested and clarified by centrifugation at 1, 000 × g for 10 min at 4 °C. The levels of interleukin-6 (IL-6) and tumor necrosis factor-alpha (TNF-α) were quantified using human ELISA kits (IL-6: R&D Systems, DY206; TNF-α: R&D Systems, DY210) following the manufacturer’s instructions. Optical density was measured at 450 nm using a microplate reader (SpectraMax iD3, Molecular Devices), and cytokine concentrations were calculated from standard curves generated with recombinant proteins provided in the kits.

### Quantitative real-time PCR

Total RNA was isolated from THP-1 cells with TRIzol reagent (Invitrogen, 15596026) according to the manufacturer’s guidelines. RNA integrity and purity were assessed using a NanoDrop 2000 spectrophotometer (Thermo Fisher Scientific). Complementary DNA (cDNA) was synthesized with the PrimeScript RT reagent kit (Takara, RR037A). Quantitative PCR was carried out with SYBR Green Master Mix (Applied Biosystems, 4367659) on a StepOnePlus Real-Time PCR System (Applied Biosystems). Expression levels of candidate genes H2BC4 and H2BC12 were normalized to GAPDH as an internal control. Relative quantification was performed using the 2^−ΔΔCt method. Primer sequences used in this study are listed in **Table 1**.

**Table 1:** Primer sequences list.

Gene	Primer	Primer sequences(5’-3’)
H2BC4	H2BC4-F	ATACGCACATACGCACAGGAT
	H2BC4-R	CGTATGGTGCGTATTGCGTA
H2BC12	H2BC12-F	GTGACTAAGGCGCAGAAGAA
	H2BC12-R	ATGCGTTCGAAGATGTCGTTG
GAPDH	GAPDH-F	AAGATCATCAGCAATGCCTCC
	GAPDH-R	AGGTTTTTCTAGACGGCAGG

### Statistical analysis

Statistical analyses were performed using IBM SPSS Statistics version 22.0 (IBM Corp., Armonk, NY, USA) and R software version 4.3.2 (R Foundation for Statistical Computing, Vienna, Austria). Data were expressed as mean ± standard deviation (SD). Group comparisons were conducted using Student’s t-test or one-way ANOVA as appropriate. A two-tailed p < 0.05 was considered statistically significant.

## Results

### Identification of differentially expressed genes in ARDS

**Figure 1:**
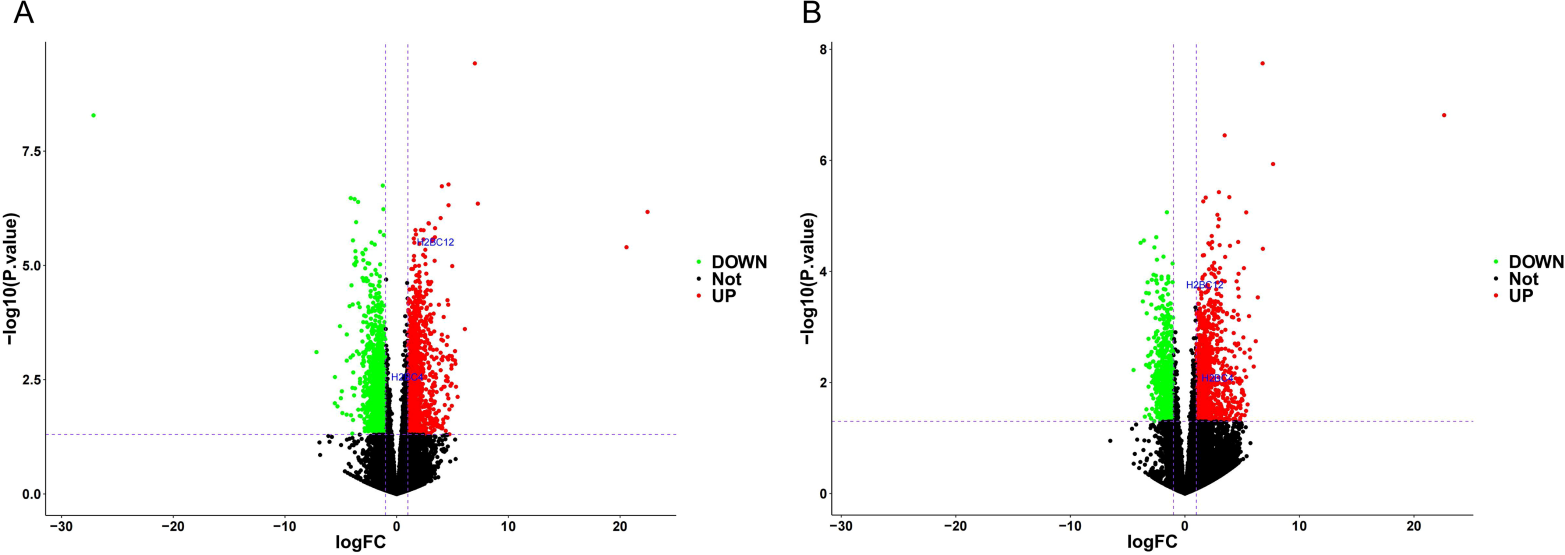
Differential expression analysis of ARDS and control samples. **(A)** Volcano plot of DEGs in the GSE200847 dataset. **(B)** Volcano plot of DEGs in the GSE163426 dataset. Red dots indicate upregulated genes, green dots indicate downregulated genes, and black dots represent non-significant genes.

To investigate transcriptional alterations in ARDS, differential expression analysis was performed using two independent GEO datasets: GSE200847 (41 ARDS patients and 5 controls) and GSE163426 (32 ARDS patients and 5 controls). In the GSE200847 cohort, a total of 2, 628 differentially expressed genes (DEGs) were identified, including 1, 324 upregulated and 1, 304 downregulated genes (**Figure 1A**). In the GSE163426 dataset, 1, 569 DEGs were detected, of which 976 were upregulated and 593 were downregulated (**Figure 1B**). Notably, two histone genes H2BC12 (HIST1H2BK) and H2BC4 (HIST1H2BC) were consistently located in the upregulated region across both datasets.

### Batch effect assessment and correction

To evaluate the presence of batch effects between GSE200847 and GSE163426, PCA was performed before and after batch correction. Prior to correction, samples exhibited partial clustering according to dataset origin, indicating the presence of batch effects. After applying batch correction, the separation between datasets was markedly reduced, and samples from different cohorts showed increased overlap in the PCA space, indicating that dataset-driven clustering was substantially reduced and cross-dataset mixing was improved after batch correction, demonstrating effective mitigation of batch effects (**Supplementary Figure 1**).

### WGCNA identifies ARDS-associated modules

**Figure 2:**
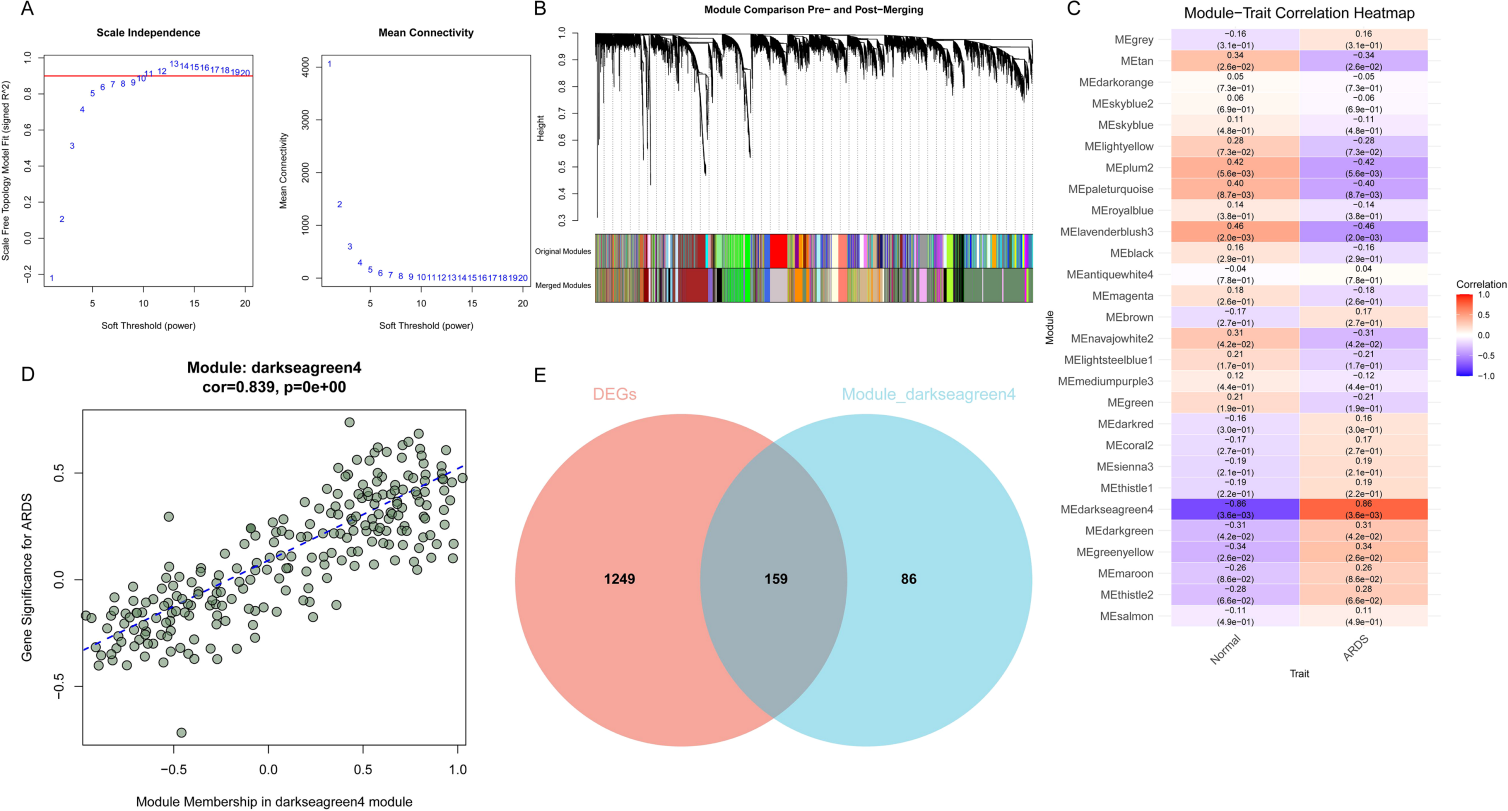
Weighted gene co-expression network analysis (WGCNA) of ARDS-associated genes. **(A)** Determination of the soft-thresholding power (β = 10) to approximate scale-free topology. **(B)** Gene dendrogram showing clustering of modules, with different colors representing distinct gene modules. **(C)** Heatmap of correlations between gene modules and ARDS clinical status. **(D)** Scatter plot of the correlation between the degree of module belongingness and the significance of genes in the darkseagreen4 module. **(E)** Venn diagram showing the overlap between DEGs and darkseagreen4 module genes, yielding 159 intersecting genes.

After batch correction and data preprocessing, a WGCNA analysis was conducted on the merged dataset containing all samples from GSE200847 and GSE163426. A total of 18, 110 genes were involved in the analysis. The soft threshold parameter β was set to 10 to achieve a scale-free topological structure (**Figure 2A**). The gene module clustering diagram identified 28 modules, excluding the gray module (which contains unassigned genes and is not considered biologically meaningful). The module–trait relationship heatmap (**Figure 2C**) displays the correlation between gene modules and ARDS status. The darkseagreen4 module has the strongest correlation with ARDS, containing 245 genes (**Figure 2D**), and this module was selected as the object for subsequent analysis. The 245 genes of the darkseagreen4 module were cross-validated with the 1, 408 differentially expressed genes previously identified, resulting in 159 intersecting genes (**Figure 2E**).

### Machine learning identifies robust candidate hub genes in ARDS

**Figure 3:**
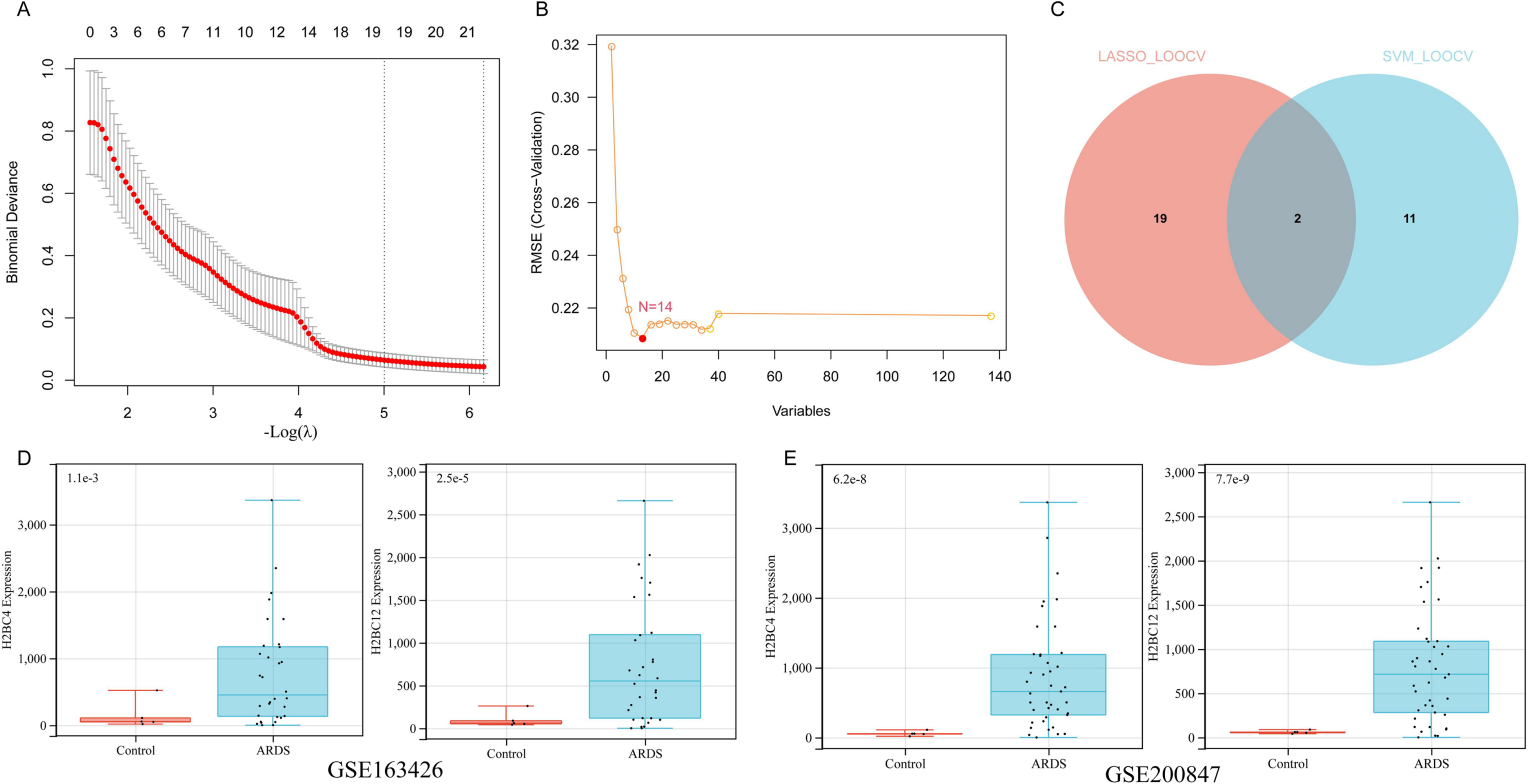
Machine learning–based screening of candidate hub genes in ARDS. **(A)** LASSO cross-validation error plot. The optimal lambda value was selected based on minimal mean squared error. **(B)** SVM-RFE feature selection results showing the number of genes retained across different feature subsets. **(C)** Venn diagram showing the intersection of genes identified by LASSO and SVM-RFE. **(D-E)** The expression of H2BC4 and H2BC12 in the GSE163426 and GSE200847 datasets.

To further screen the candidate genes, a machine learning algorithm analysis was conducted on the 159 overlapping genes identified from the intersection of DEGs and WGCNA module genes. Using LASSO regression with leave-one-out cross-validation (LOOCV) for feature selection, 21 genes were identified based on the optimal lambda value (**Figure 3A**). The SVM-RFE analysis using the e1071 package screened out 14 genes (**Figure 3B**). The intersection of lasso regression and SVM support vector machine yielded 2 genes, H2BC4 and H2BC12 (**Figure 3C**). Subsequently, the expressions of these two genes in the GSE163426 and GSE200847 datasets were analyzed, and a consistent differential expression pattern was observed in the ARDS group and the control group samples (**Figures 3D, E**).

### Functional enrichment analysis reveals key biological processes associated with ARDS

**Figure 4:**
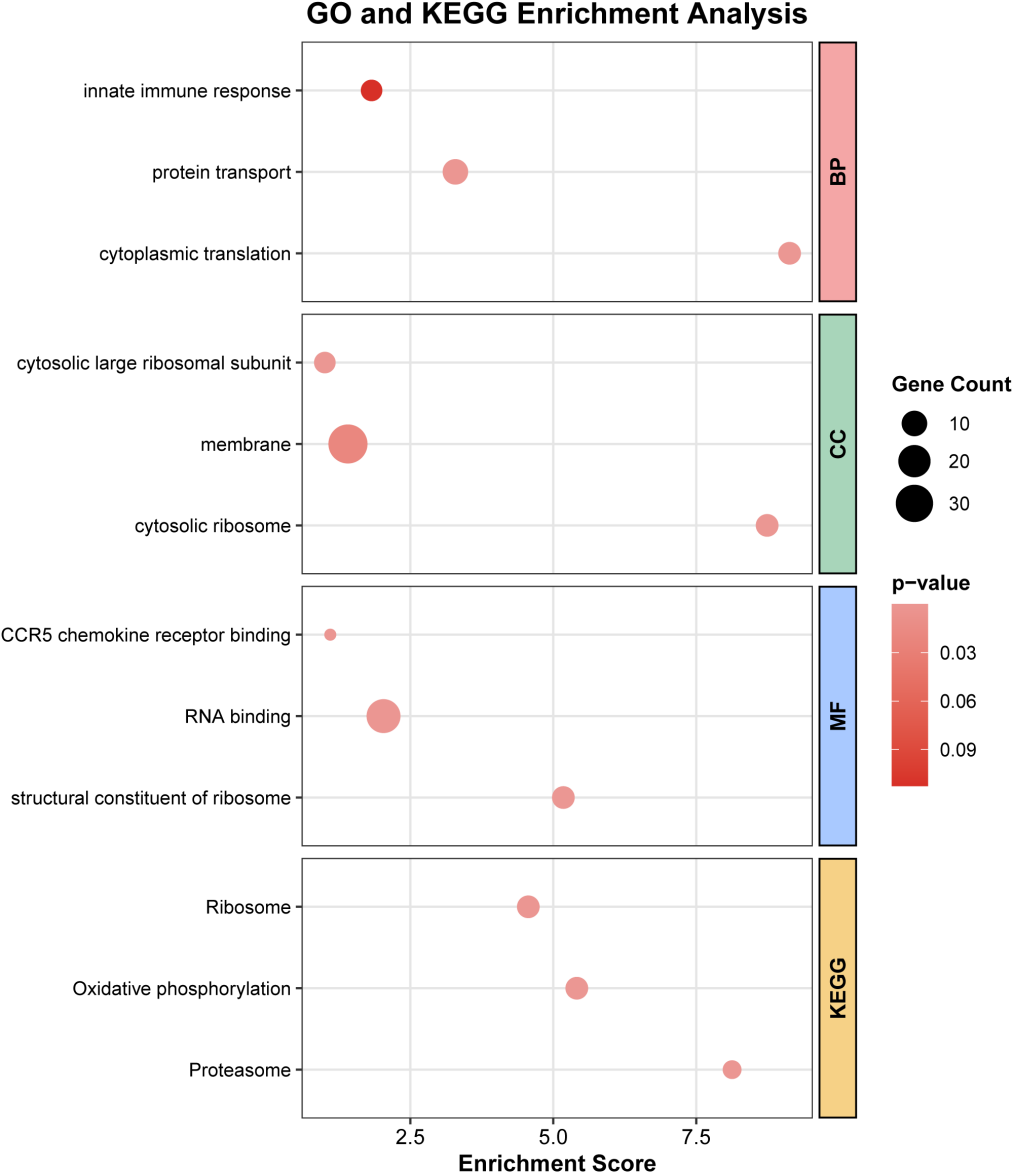
GO and KEGG functional enrichment analysis of the 245 genes from the WGCNA darkseagreen4 module.

To explore the biological functions of the identified key genes, we used the clusterProfiler software package to conduct an enrichment analysis on 245 genes from the darkseagreen4 module obtained from the WGCNA analysis. A total of 9 GO terms and 3 KEGG terms were obtained (**Figure 4**). The module genes were mainly enriched in ribosome/translation-related structures and functions, innate immune response, protein transport, oxidative phosphorylation, and the proteasome pathway. Among them, the GO cellular components were mainly located in cytosolic large ribosomal subunit, cytosolic ribosome, and membrane, while the biological processes mainly involved innate immune response, cytoplasmic translation, and protein transport. The molecular functions mainly included structural component of ribosome, RNA binding, and CCR5 chemokine receptor binding; the KEGG pathways were mainly enriched in Oxidative phosphorylation, Ribosome, and Proteasome. Overall, it suggests that the changes of these differentially expressed genes mainly occurred in protein synthesis and processing, energy metabolism, and immune inflammatory-related processes.

### Immune infiltration analysis reveals distinct immune alterations in ARDS

**Figure 5:**
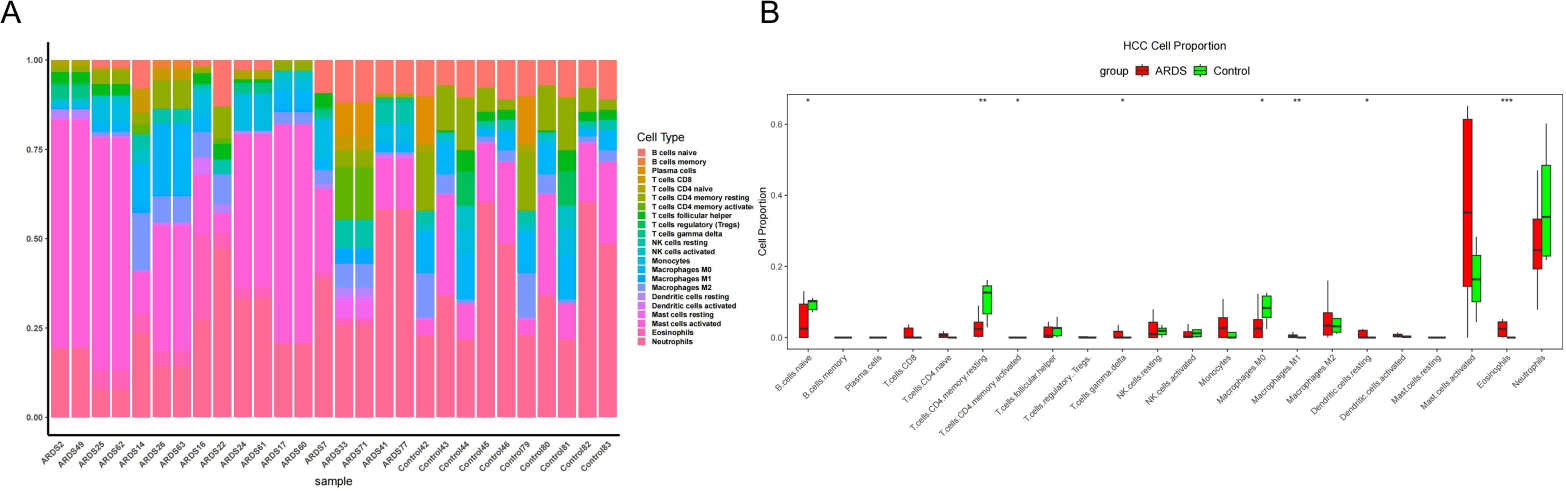
Immune infiltration profiles in ARDS and controls inferred by CIBERSORT. **(A)** Stacked bar plot showing the relative fractions of 22 immune cell subsets across ARDS and control samples. **(B)** Boxplots comparing immune cell proportions between ARDS and controls. Statistical significance was determined using Wilcoxon rank-sum tests with Benjamini-Hochberg correction for multiple comparisons.

CIBERSORT-based deconvolution of the merged bulk RNA-seq data revealed the relative abundance of 22 immune cell subsets across ARDS and control samples (**Figure 5A**). The overall immune landscape demonstrated marked heterogeneity among individuals, with myeloid-derived subsets generally dominating the cellular composition. Comparative analysis between groups identified significant alterations in multiple immune populations after adjustment for multiple testing (FDR < 0.05) (**Figure 5B**). Specifically, naïve B cells were reduced in ARDS, whereas resting CD4 memory T cells were markedly decreased and activated CD4 memory T cells were increased. γδ T cells also showed a moderate increase. Within the myeloid compartment, macrophages M0 were decreased, while M1 macrophages were significantly increased. Resting dendritic cells were more abundant in ARDS, whereas eosinophils were markedly reduced.

### Single-cell transcriptome analysis validates cell-specific expression of hub genes

**Figure 6:**
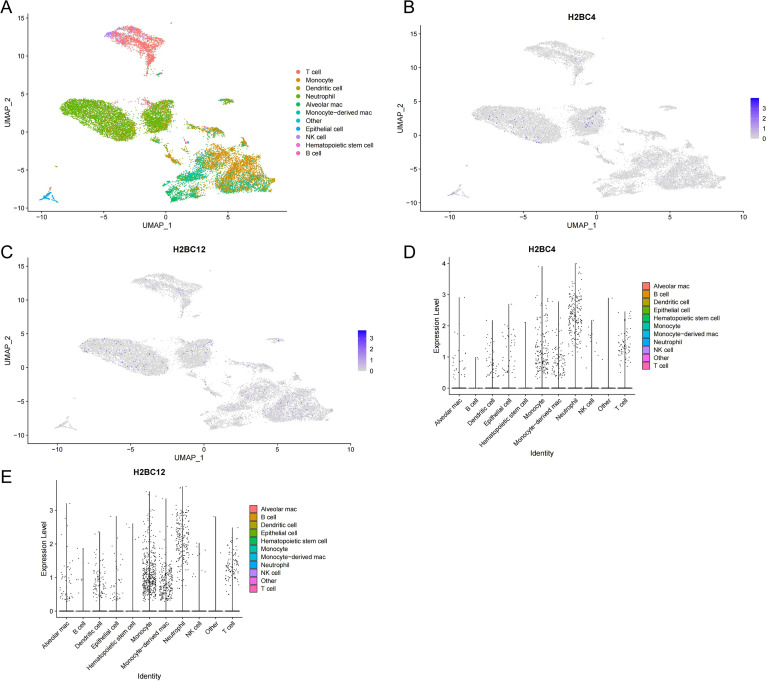
Single-cell transcriptome validation of hub gene expression in ARDS. **(A)** UMAP plot showing clustering of 18, 717 cells into distinct immune and non-immune populations. **(B, C)** UMAP feature plots displaying the expression of H2BC4 and H2BC12 across cell clusters. **(D, E)** Violin plots illustrating expression levels of H2BC4 and H2BC12 in different cell types.

To localize the expression of candidate hub genes at the single-cell level, the GSE200848 dataset comprising 18, 717 high-quality cells from 8 ARDS patients was analyzed using the Seurat package. UMAP clustering distinguished multiple immune and non-immune cell populations, including T cells, B cells, NK cells, neutrophils, monocytes, macrophages, dendritic cells, and epithelial cells (**Figure 6A**). Expression mapping demonstrated that both H2BC4 and H2BC12 were predominantly expressed in myeloid-derived populations, including neutrophils, monocytes, and macrophages, while showing minimal expression in lymphoid subsets such as T cells, B cells, and NK cells (**Figures 6B, C**). Violin plots further confirmed the high expression of H2BC4 and H2BC12 in myeloid cells, in contrast to their low expression in adaptive immune lineages (**Figures 6D, E**).

### Experimental validation of candidate genes in LPS-induced THP-1 cells

**Figure 7:**
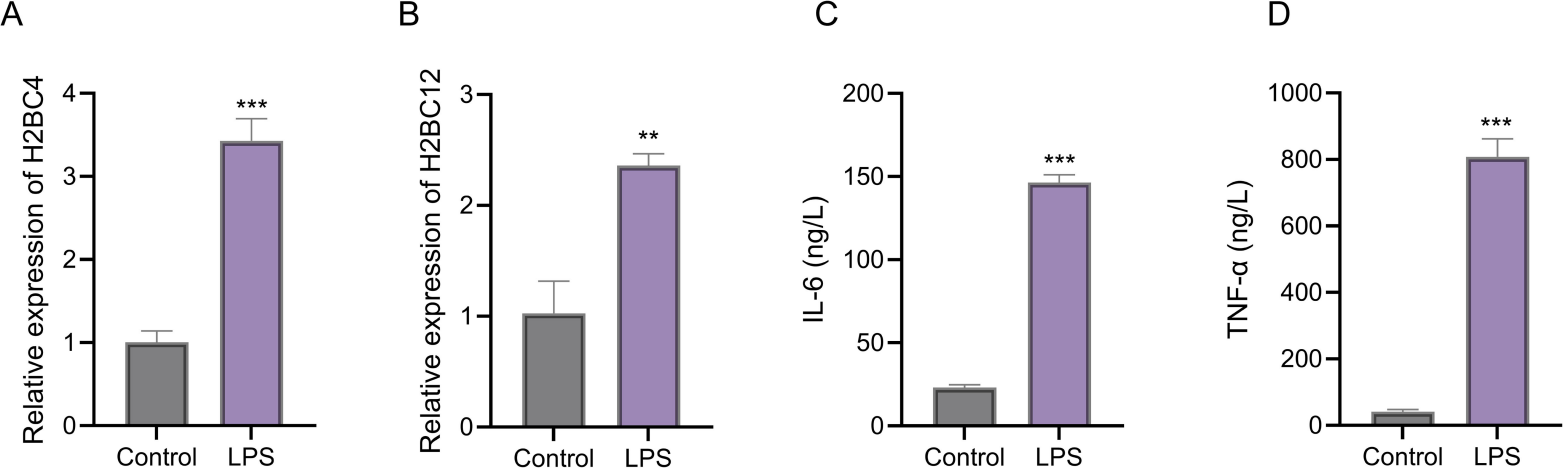
Experimental validation of hub gene expression and cytokine production in LPS-induced THP-1 cells. **(A, B)** RT-qPCR analysis of H2BC4 and H2BC12 expression in control and LPS-treated THP-1 cells. **(C, D)** ELISA quantification of IL-6 and TNF-α levels in culture supernatants from control and LPS-treated cells.

To validate the computational predictions, RT-qPCR and ELISA assays were performed in an LPS-induced THP-1 inflammatory model. RT-qPCR analysis showed that H2BC4 and H2BC12 expression was extremely low or undetectable in control cells, whereas both genes were significantly upregulated upon LPS stimulation (p<0.01; **Figures 7A, B**). Consistently, ELISA measurements revealed that secretion of IL-6 and TNF-α was minimal under control conditions but markedly increased after LPS treatment (p<0.001; **Figures 7C, D**).

## Discussion

In this study, we systematically characterized the immune imbalance landscape of ICU patients with ARDS by integrating bulk transcriptomic data, network analysis, machine learning, and single-cell transcriptomics, followed by *in vitro* validation. Our integrative analysis identified two histone-related genes, H2BC4 and H2BC12, as candidate hub genes associated with ARDS. These genes exhibited preferential expression in myeloid populations and were significantly upregulated under inflammatory stimulation, providing novel insights into the molecular basis of immune dysregulation in ARDS.

While previous transcriptomic studies have primarily focused on cytokines, chemokines, and classical inflammatory mediators in ARDS, our findings extend this framework by identifying histone-related genes as potential contributors (11). Notably, H2BC4 and H2BC12 have not been previously reported in ARDS. However, emerging evidence from other disease contexts supports a broader role for H2B family members in cellular stress responses. For instance, Ou et al. (2025) (12) reported H2bc4 upregulation in spermatogonial stem cells under epigenetic stress, and Hade et al. (2025) (13) identified H2BC12 enrichment in extracellular vesicles from obese patients. These convergent observations suggest that H2BC4 and H2BC12 may function as general stress-responsive regulators. Importantly, our study provides the first evidence linking these genes to ARDS and demonstrates their enrichment in myeloid cells and inducibility under inflammatory conditions, thereby revealing a previously underappreciated role of chromatin-related mechanisms in ARDS pathogenesis.

Functional enrichment analysis further revealed that ARDS-associated genes are primarily involved in pathways related to protein synthesis, energy metabolism, and innate immune processes. Specifically, GO terms such as cytoplasmic translation and protein transport, along with KEGG pathways including oxidative phosphorylation, ribosome, and proteasome, were significantly enriched. These findings suggest that activated immune cells in ARDS undergo substantial metabolic reprogramming and enhanced protein turnover to sustain their functional demands. Such processes are essential for supporting rapid cytokine production and immune activation, and are consistent with previous studies highlighting the importance of metabolic adaptation in inflammatory responses (14, 15).

Consistent with these pathway-level findings, immune cell deconvolution analysis demonstrated a marked shift toward myeloid-dominated immune responses in ARDS. Specifically, increased proportions of M1 macrophages and dendritic cells, along with elevated γδ T cells and reduced naïve B cells, resting CD4 memory T cells, and eosinophils, indicate profound immune remodeling (16, 17). These changes reflect a transition toward a pro-inflammatory state characterized by enhanced innate immune activation and impaired adaptive immune regulation. Particularly noteworthy is the increased presence of mast cells, which have received limited attention in ARDS but may contribute to vascular permeability and tissue injury (18).

Single-cell transcriptomic analysis was used to test the hypothesis generated from bulk analyses and provided further resolution at the cellular level, demonstrating that H2BC4 and H2BC12 are predominantly expressed in myeloid-derived populations, including neutrophils, monocytes, and macrophages. This cell-type specificity supports the hypothesis that these genes are involved in innate immune activation rather than adaptive immune processes. Furthermore, *in vitro* experiments using LPS-stimulated THP-1 cells confirmed that both genes are inducible under inflammatory conditions, providing functional support for their potential role in ARDS-related immune responses. Together, these findings suggest that H2BC4 and H2BC12 may contribute to the amplification of innate immune signaling in ARDS.

Building on these observations, the identification of histone-related genes as candidate mediators raises important mechanistic implications. As core components of nucleosomes, H2BC4 and H2BC12 are involved in regulating chromatin accessibility and transcriptional activity. Their upregulation in myeloid cells may facilitate the activation of pro-inflammatory transcriptional programs, thereby enhancing cytokine production and sustaining inflammatory responses. This epigenetic regulation may contribute to the exaggerated inflammatory milieu and impaired resolution characteristic of ARDS (14, 15). Clinically, these findings suggest that H2BC4 and H2BC12 may serve as potential biomarkers for immune dysregulation and may represent novel therapeutic targets. Modulation of chromatin dynamics could therefore provide a complementary strategy to existing anti-inflammatory approaches in ARDS.

Several limitations of this study should be acknowledged. First, the transcriptomic datasets were derived from publicly available cohorts with relatively limited sample sizes, which may introduce heterogeneity despite batch correction. Second, we employed a conservative intersection-based strategy to identify differentially expressed genes across datasets; although this approach enhances robustness, it may overlook genes with moderate but consistent effects. Future studies incorporating larger cohorts and formal meta-analysis methods, such as Fisher’s combined p-values or Stouffer’s Z-score method, may improve sensitivity and statistical power. Third, experimental validation was limited to an *in vitro* model using THP-1 cells, and further validation in independent clinical cohorts and *in vivo* models is required. Fourth, the downstream molecular mechanisms regulated by H2BC4 and H2BC12 remain unclear and warrant further investigation. Finally, ARDS subphenotypes were not incorporated into the WGCNA analysis, which may limit the identification of subtype-specific molecular signatures. Future studies integrating multi-omics approaches, including proteomics and epigenomics, will be essential to further elucidate the regulatory networks underlying ARDS and to facilitate translation into precision medicine.

## Conclusion

This study provides new insights into the immunopathogenesis of ARDS by integrating transcriptomic analyses with machine learning, single-cell validation, and experimental assays. The findings highlight potential molecular targets that may contribute to the development of immune-based diagnostic and therapeutic strategies. Together, our work underscores the importance of systematically characterizing immune dysregulation to inform future precision medicine approaches in ARDS.

## Data Availability

The original contributions presented in the study are included in the article/**Supplementary Material**. Further inquiries can be directed to the corresponding authors.
