# Supplementary material for: Transcriptomic analysis reveals immune dysregulation and identifies key genes in ICU patients with severe ARDS
Source: Front Immunol. 2026 May 11;17:1777938. doi: 10.3389/fimmu.2026.1777938 (PMC13199009; doi:10.3389/fimmu.2026.1777938)
Supplement: Supplementary file 1 [file DataSheet1.docx]

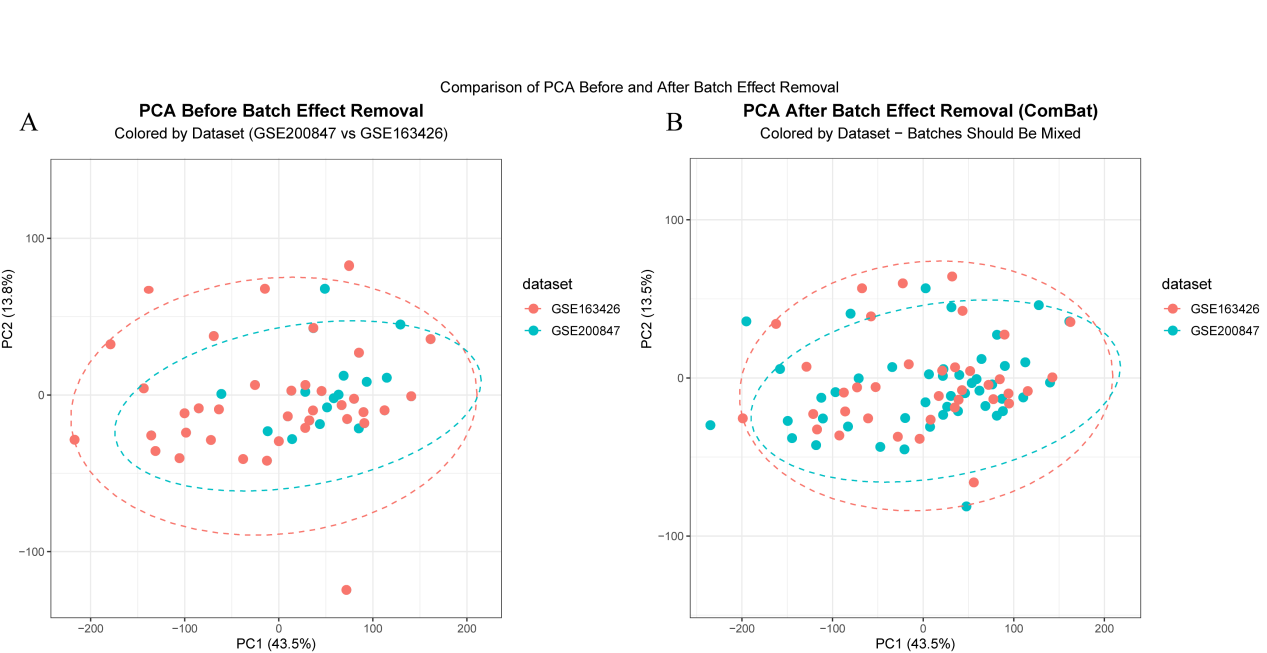


Supplementary Figure 1. PCA analysis before and after batch correction.

(A) PCA plot before batch correction showing clustering of samples by dataset origin (GSE200847 vs. GSE163426).

(B) PCA plot after batch correction demonstrating improved mixing of samples and reduced batch effects.

Supplementary Table 1.KEGG enrichment pathway-related genes.

| Pathway | Genes |
| --- | --- |
| Ribosome | RPL28; RPS15A; RPL26; UBA52; RPL37; RPL32; RPL35A |
| Oxidative phosphorylation | ATP6V1D; NDUFA4; ATP5F1C; COX7A2L; ATP5PB; ATP6V1E1; NDUFB1 |
| Proteasome | PSMC4; PSMA4; PSMA6; PSMB8 |
